# Blood Transcriptomics of Turbot *Scophthalmus maximus*: A Tool for Health Monitoring and Disease Studies

**DOI:** 10.3390/ani11051296

**Published:** 2021-04-30

**Authors:** Paolo Ronza, José Antonio Álvarez-Dios, Diego Robledo, Ana Paula Losada, Roberto Romero, Roberto Bermúdez, Belén G. Pardo, Paulino Martínez, María Isabel Quiroga

**Affiliations:** 1 Departamento de Anatomía, Producción Animal y Ciencias Clínicas Veterinarias, Facultad de Veterinaria, Universidade de Santiago de Compostela, 27002 Lugo, Spain; anapaula.losada@usc.es (A.P.L.); roberto.bermudez@usc.es (R.B.); misabel.quiroga@usc.es (M.I.Q.); 2 Departamento de Matemática Aplicada, Facultad de Matemáticas, Universidade de Santiago de Compostela, 15782 Santiago de Compostela, Spain; joseantonio.alvarez.dios@usc.es; 3 The Roslin Institute and Royal (Dick) School of Veterinary Studies, University of Edinburgh, Midlothian EH25 9RG, UK; diego.robledo@roslin.ed.ac.uk; 4 Insuiña SL, Playa del Lago, 27870 Xove, Spain; rromero@nuevapescanova.com; 5 Instituto de Acuicultura, Universidade de Santiago de Compostela, 15782 Santiago de Compostela, Spain; belen.gomez@usc.es (B.G.P.); paulino.martinez@usc.es (P.M.); 6 Departamento de Zoología, Genética y Antropología Física, Facultad de Veterinaria, Universidade de Santiago de Compostela, 27002 Lugo, Spain

**Keywords:** RNA-seq, teleost, immune system, immune response, erythrocytes, leukocytes, *Enteromyxum scophthalmi*, infection, myxozoa

## Abstract

**Simple Summary:**

The analysis of blood gene expression is emerging as a relevant source of information about the health status of an organism. While these investigations are increasingly performed in human and terrestrial animals, their potential is still underexplored in fish pathology. The aim of this work was to analyze the blood transcriptional profile of a commercially important flatfish species, turbot (*Scophthalmus maximus*), in healthy and diseased specimens. The analysis of the most expressed genes in healthy fish indicated that turbot red blood cells have important immunological functions. In diseased fish, parasitized by a myxozoan, the blood analysis reflected a broad inhibition of the immune response followed by intense inflammatory activation in heavy infections. The results showed that turbot response appears delayed, dysregulated and ineffective in stopping the infection. Particularly, a proper development of the adaptive immune response was lacking. This study points out that blood gene expression profiling is a reliable tool for health monitoring, as well as to advance in the knowledge of fish immunity and diseases.

**Abstract:**

Blood transcriptomics is emerging as a relevant tool to monitor the status of the immune system and assist in diagnosis, prognosis, treatment and pathogenesis studies of diseases. In fish pathology, the potential of transcriptome profiling of blood is still poorly explored. Here, RNA sequencing was applied to analyze the blood transcriptional profile of turbot (*Scophthalmus maximus*), the most important farmed flatfish. The study was conducted in healthy specimens and specimens parasitized by the myxozoan *Enteromyxum scophthalmi*, which causes one of the most devastating diseases in turbot aquaculture. The blood of healthy turbot showed a transcriptomic profile mainly related to erythrocyte gas transportation function, but also to antigen processing and presentation. In moderately infected turbot, the blood reflected a broad inhibition of the immune response. Particularly, down-regulation of the B cell receptor signaling pathway was shared with heavily parasitized fish, which showed larger transcriptomic changes, including the activation of the inflammatory response. Turbot response to enteromyxosis proved to be delayed, dysregulated and ineffective in stopping the infection. The study evinces that blood transcriptomics can contribute to a better understanding of the teleost immune system and serve as a reliable tool to investigate the physiopathological status of fish.

## 1. Introduction

Blood is a major component of the immune system and acts as a pipeline carrying leukocytes and humoral factors throughout the body [1]. The ease of sampling, minimal invasiveness and its informativeness regarding animal homeostasis are qualities that crown it a highly desirable tissue for disease prediction, diagnosis, monitoring, prognosis and biomarker development [2]. Blood is routinely employed to monitor the state of the immune system by hematological analyses in mammals. Although hematology is also considered a valuable diagnostic tool in fish, it is still of limited use in that context given the lack of reference values [3]. This is also due to the broad differences observed among species as well as the several physiological variables that can affect blood parameters within species (reproductive cycle, age, sex, feeding behavior, stress, nutritional status, habitat or culture system, water quality) [4].

Beyond the foreseeable standardization of hematology in fish health monitoring, the progress of next-generation high-throughput sequencing technology has opened new avenues to study what blood can reveal about the physiopathological status of an organism [2,5]. As molecular processes driving complex diseases usually involve sets of genes acting in a concerted way, one strategy is to use high-throughput “omics” technologies to simultaneously investigate thousands of genes in a pathological context [6]. In human medicine, it has been observed that the analysis of the blood transcriptome can provide a comprehensive view of the state of the immune system, advance our understanding of disease pathogenesis and highlight potential biomarkers to be used in diagnosis, prognosis and treatment monitoring [5,7,8].

RNA sequencing (RNA-Seq)-based transcriptome analysis is increasingly used to better understand host-pathogen interactions in fish. Transcriptomic profiles have been obtained from many fish tissues in several studies. Most of them deal with the analysis of the main lymphoid organs (kidney and spleen) or mucosal tissues in organs often targeted by pathogens (gill, digestive tract and skin) [9]. However, few researchers have focused on fish blood transcriptome [10], and most of the recent studies have been mainly focused on the role of fish nucleated red blood cells in viral diseases [11,12,13,14].

In turbot (*Scophthalmus maximus*), a marine flatfish of high commercial value, RNA-Seq analysis was previously applied to gain insights into the host–parasite interaction during enteromyxosis. This disease, which poses a serious threat to turbot aquaculture, is caused by the myxozoan parasite *Enteromyxum scophthalmi* [15], included in the EU H2020-funded project ParaFishControl as one of the most harmful parasitic species affecting the main European farmed teleosts (https://www.parafishcontrol.eu/, accessed on 16 April 2021). The transcriptomic profiles of the major lymphohematopoietic organs (kidney, spleen and thymus) and the main target organ (digestive tract) have been analyzed [15,16]. Nevertheless, the blood transcriptome has not been evaluated during enteromyxosis before, nor, to our knowledge, ever studied in this species. The identification of non-lethal biomarkers for early diagnosis and/or treatment development would greatly help to gain control of this parasitosis, against which there are currently no therapeutic options [15].

In this work, we carried out a transcriptomic analysis of blood tissue in *S. maximus*, studying healthy and *E. scophthalmi*-infected specimens. RNA-Seq was performed by using an Illumina NovaSeq platform, and the sequencing reads were mapped to the turbot reference genome [17]. Afterwards, differentially expressed genes (DEGs) were identified, and a functional enrichment analysis was performed. The results provide valuable insights into the blood transcriptome of turbot in health and disease, and constitute a useful resource for further research into the defense mechanisms of teleosts.

## 2. Materials and Methods

### 2.1. Experimental Design

Turbot (*n* = 280, 200 g mean weight) were obtained from a farm in Galicia (Northwestern Spain) and kept at the facilities of CETGA (Aquaculture Cluster Technology Centre, Ribeira, A Coruña, Spain). For experimental purposes, animals were divided into 130 control and 150 recipient fish. They were acclimated for one week under identical conditions before starting the trial in 12 tanks with 5 μm-filtered and UV-irradiated open-flow sea water at environmental temperature. The experimental infection was carried out by oral route, as described by Redondo et al. [18]. Recipient fish received 1 mL of intestinal scraping homogenates in Hank’s Balanced Salt Solution (HBSS) from *E. scophthalmi*-infected donor fish, whereas control fish were inoculated with the same amount of HBSS alone. Donor turbot came from a natural outbreak in a turbot farm. 15 recipient and 10 control fish were sampled at 7, 20, 50 and 79 days post-inoculation (dpi). At each sampling point, fish were sedated with tricaine methanesulphonate at a dose of 70 mg/L (Tricaine PHARMAQ 1000 mg/g, PHARMAQ, Overhalla, Norway), their weight and length were recorded, and blood samples were taken. After that, individuals were euthanized with the same anesthetic, at a dose of 500 mg/L, and necropsied to obtain tissue samples.

### 2.2. Sampling

Blood was obtained from the caudal vein and blood smears were immediately prepared for diagnostic purposes. Subsequently, about 1 mL of blood per fish was collected in EDTA tubes and gently mixed. For transcriptomic analysis, a total of 400 μL from this blood was transferred to a tube containing 1.3 mL of RNAlater (Ambion; Thermo Fisher Scientific, Waltham, MA, USA) and thoroughly mixed by pipetting. The rest of the blood was employed for PCR-based diagnosis. Both sample types were kept refrigerated until arrival at the laboratory, where they were stored at a temperature of −20 °C. Blood smears were allowed to air dry before being fixed in ice-cold acetone for 5 min. Then, one smear per fish was stored in slide boxes to be used for immunocytochemical assays and one was stained with Diff Quick^®^ (Química Clínica Aplicada, Amposta, Spain) for cytologic examination.

At necropsy, small pieces of pyloric caeca were taken in 1 mL ethanol 100% for PCR-based diagnosis before extracting the visceral organs from the coelomic cavity. Tissue samples from the different organs were fixed in 10% neutral buffered formalin for the histopathological evaluation.

### 2.3. Histopathological, Immunochemical and PCR-Based Diagnosis

Formalin-fixed samples were processed for paraffin-embedding and then thin sections thereof (3 μm) were stained with haematoxylin and eosin and toluidine blue for microscopic evaluation. The healthy status of control fish was checked, and *E. scophthalmi*-challenged fish were evaluated according to the histopathologic grading described by Bermúdez et al. [19].

A monoclonal antibody against *E. scophthalmi* (kindly donated by Dr. R. Iglesias and Dr. J.M. García-Estevez, Universidade de Vigo, Spain) was employed to carry out the detection of *E. scophthalmi* by immunohistochemistry (IHC) and immunocytochemistry (ICC). For IHC, thin sections from paraffin-embedded samples of the digestive tract were placed onto slides treated with silane, to improve section adherence, and dried overnight at 37 °C. Endogenous peroxidase activity was quenched by incubation with a peroxidase-blocking solution (BLOXALL, Vector Laboratories, Burlingame, CA, USA), then the slides were incubated for 2 h at room temperature with the monoclonal antibody (1:5000 working dilution). A commercial kit (ImmPRESS^®^ VR Anti-Mouse IgG HRP Polymer Detection Kit, Vector Laboratories, Burlingame, CA, USA) including a peroxidase (HRP)-labelled polymer conjugated to mouse secondary antibody was employed following the manufacturer’s instructions, and finally the peroxidase reaction was developed using a diaminobenzidine-positive chromogen (Dako, Glostrup, Denmark). All incubations were performed in a humid chamber at room temperature. The sections were counterstained with hematoxylin, dehydrated and coverslipped with DePeX mounting medium. Three 5 min washes in 0.1 M phosphate buffered saline (PBS) containing 0.05% Tween-20 were executed between all subsequent steps. Positive controls consisted of samples from naturally infected fish, while negative controls included samples from uninfected fish and sections where the primary antibody was replaced by PBS. ICC was performed on blood smears. In this case, after the quenching of endogenous peroxidase activity, the slides were incubated for 1 h at room temperature with the primary antibody at the working dilution of 1:50,000; afterwards, the subsequent steps were the same described for IHC.

For PCR-based diagnosis, genomic DNA from blood was extracted and purified with the DNeasy Blood & Tissue kit (Qiagen, Manchester, UK). DNA from pyloric caeca was extracted with Chelex^®^100 resin (Bio-Rad, Hercules, CA, USA). DNA samples were stored at −20 °C until use. The Internal transcribed spacer 1 (ITS-1) sequence of *E. scophthalmi* was targeted for PCR-based diagnosis. A region of 162 bp of ITS-1 was amplified using forward primer ES-1F 5′-CCACACACCCACCAAAGTGT-3′ and reverse primer ES-1R 5′-ACGTCTAGCACCCATCCTTC-3′. PCR reactions (50 µL) included 1X PCR Gold Buffer (Applied Biosystems, Foster City, CA, USA), 1.25 mM of MgCl_2_, 200 µM of dNTPs, 0.1 µM of each primer, 5 units of Amplitaq Gold™ DNA polymerase (Applied Biosystems) and 100 ng of blood DNA or 1.5 µL of Chelex DNA as template. PCR was performed in a Veriti Thermal Cycler (Applied Biosystems, Foster City, CA, USA) as follows: an initial denaturation at 95 °C for 10 min followed by 35 cycles at 94 °C for denaturation for 45 s, 50 s at 62 °C and 50 s at 72 °C for extension. A final extension step was performed at 72 °C for 10 min. PCR products (7 µL) were run on a 1% agarose gel.

### 2.4. RNA-seq

RNA was isolated using the RiboPure-Blood kit (Ambion-Thermo Fisher Scientific, Waltham, MA, USA) with DNase treatment following the manufacturer’s instructions. RNA quality and quantity were evaluated in a Bioanalyzer (Bonsai Technologies, Bhubaneswar, India) and in a NanoDrop^®^ ND-1000 spectrophotometer (NanoDrop^®^ Technologies Inc., Wilmington, DE, USA), respectively. Samples from control fish were pooled, for a final amount of four samples to be sent for RNA-sequencing. On the other hand, infected fish were analyzed individually. Samples were barcoded and prepared for sequencing by Edinburgh Genomics (Edinburgh, UK) on Illumina NovaSeq S2 (Illumina, San Diego, CA, USA) as 100 bp paired-end reads. Raw sequencing data have been deposited in NCBI’s Short Read Archive (SRA) under BioProject ID PRJNA703783.

Turbot transcriptome annotations and gene names were extracted from Ensembl (http://www.ensembl.org, accessed on 10 February 2020) using BioMart (http://www.biomart.org/, accessed on 10 February 2020). RNA-seq output files were processed with Kallisto (https://pachterlab.github.io/kallisto/, accessed on 10 February 2020), taking paired-end information into account, in order to quantify expression. Resulting quantity files were summed up in a spreadsheet to characterize genes having expression levels with a high standard deviation and/or coefficient of variation. The Sleuth package (https://pachterlab.github.io/sleuth/, accessed on 3 March 2020) was used within R (https://www.r-project.org/, accessed on 3 March 2020) in order to extract lists of DEGs (FDR < 10%) and their fold change according to different group conditions, using the Likelihood Ratio Test (LTR). Final results were condensed into spreadsheets using a custom Perl script.

Kyoto Encyclopedia of Genes and Genomes (KEGG) enrichment analyses were performed using KOBAS v3.0.3 [20]. KEGG enrichments for specific gene lists were tested by comparison to the whole transcriptome of the turbot using Fisher’s Exact Test, and those terms or pathways showing a Benjamini–Hochberg FDR-corrected *p*-value < 0.05 were considered to be enriched. Protein–protein interaction networks were constructed using STRING v11 [21] with default parameters to further investigate the relationship of DEG subsets.

## 3. Results

### 3.1. Diagnosis of Enteromyxosis

**Figure 1:**
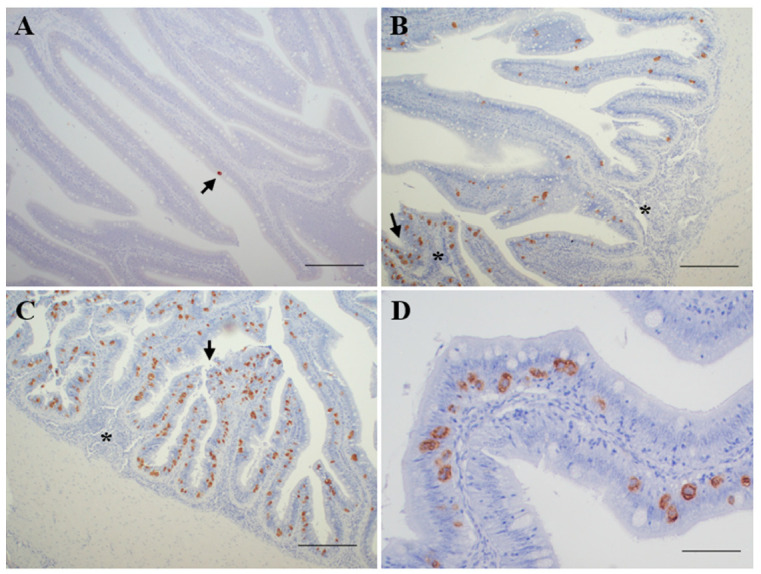
Immunohistochemical detection of *Enteromyxum scophthalmi* in gut histological sections of turbot *Scophthalmus maximus*. (**A**) Intestinal folds of a slightly infected fish. Only one parasitic form can be observed (arrow) and the specimen showed no significant histopathological lesions. Bar = 200 μm. (**B**) Intestinal folds of a turbot with moderate infection, with several immunolabeled parasites. Some areas showed higher parasite burden (arrow) and presence of inflammatory infiltrates (asterisks). Bar = 200 μm. (**C**) Intestinal folds of a severely infected turbot. Note the high parasite burden, presence of inflammatory infiltrates (asterisk) and signs of epithelial detachment (arrow). Bar = 200 μm. (**D**) High magnification of a heavily parasitized intestinal fold where several immunostained forms of *E. scophthalmi* can be observed. Bar = 50 μm.

According to the results obtained from the application of histological, immunohistochemical (Figure 1) and PCR-based techniques on digestive tract samples, the recipient fish were classified into five levels of infection:

Not infected (*n* = 8): negative detection by all the methods applied.
Incipient infection (*n* = 14): only positive by PCR diagnosis.
Slight infection (*n* = 15): positive by PCR and immunohistochemical diagnosis, showing reduced parasite burden and none or minimal histopathological lesions.
Moderate infection (*n* = 13): positive by PCR, immunohistochemical and histological diagnosis, showing signs of enteritis and most of the intestinal folds parasitized, with variable parasite burden.
Severe infection (*n* = 10): positive by PCR, immunohistochemical and histological diagnosis, showing high parasite burden and evident lesions of catarrhal enteritis.

The presence of *E. scophthalmi* was not detected in any blood sample by any of the techniques applied.

### 3.2. Blood Transcriptome of Healthy Turbot

**Figure 2:**
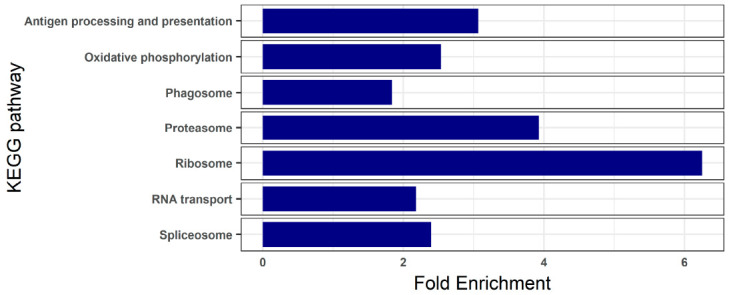
Bar graphs of significantly enriched Kyoto Encyclopedia of Genes and Genomes (KEGG) pathways in the blood of healthy turbot tested by comparison to the whole turbot transcriptome. The blood transcripts with a TPM (transcripts per million) value > 50 were tested. KEGG pathways showing a Benjamini-Hochberg FDR corrected *p*-value < 0.05 were considered to be enriched.

To understand the basal status of the turbot blood, we analyzed the transcriptomes of the control samples. A total of 1107 genes showed an expression value > 50 transcripts per million (TPM) in any of the four samples analyzed (Table S1). The most significantly enriched functions revealed by KEGG pathway enrichment analysis were related to ribosome, oxidative phosphorylation, RNA transport, spliceosome, proteasome, phagosome and antigen processing and presentation (Figure 2; Table S1).

The top three genes, corresponding to alpha and beta chains of hemoglobin, represented over 60% of the expressed transcripts. Among the top 50 genes, we also found *ferritin*, major histocompatibility complex class I (MHC-I) molecules, *immunoglobulin light chain* and several ribosome-related transcripts (Table 1).

### 3.3. Blood Transcriptome during Enteromyxosis

Differentially expressed genes were only detected in fish with moderate and severe infection. Principal component analysis based on transcriptomic data is shown in Figure S1. A total of 244 DEGs were found in fish with moderate infection (32 up- and 212 down-regulated), while those severely infected showed 957 DEGs (582 up- and 375 down-regulated) (Table S2).

**Figure 3:**
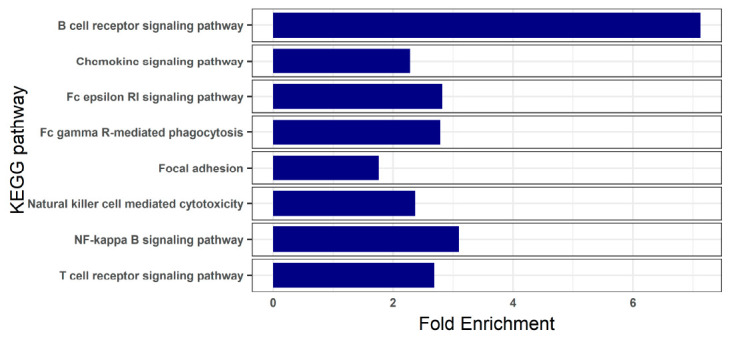
Bar plots of significantly enriched KEGG pathways in turbot blood with moderate infection by *Enteromyxum scophthalmi*. The enrichment analysis was conducted on the set of down-regulated genes found by comparison with healthy specimens. KEGG pathways showing a Benjamini-Hochberg FDR corrected *p*-value < 0.05 were considered to be enriched.

In moderate infection, KEGG pathway enrichment analyses showed no enriched functions in the set of up-regulated genes, while different signaling pathways of the immune response were enriched when analyzing down-regulated genes, such as B cell receptor, T cell receptor, chemokine and NF-kappa B signaling pathways (Figure 3; Table S2).

**Figure 4:**
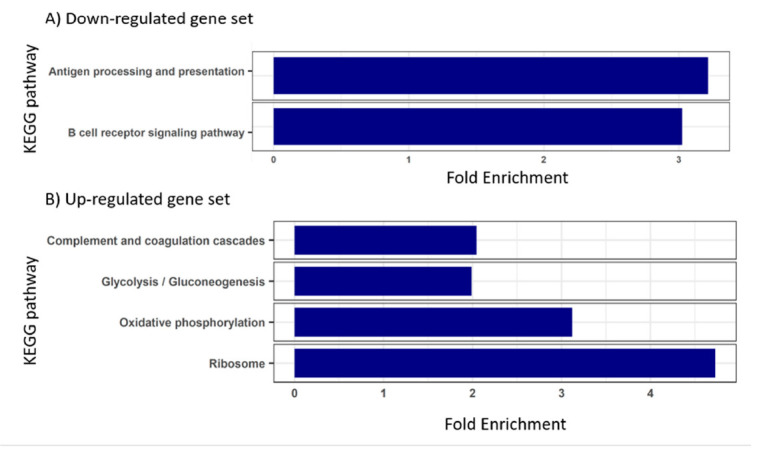
Bar plots of significantly enriched KEGG pathways in turbot blood at advanced stages of infection by *Enteromyxum scophthalmi*. The enrichment analyses were conducted on the sets of down—(**A**) and up—(**B**) regulated genes found by comparison with healthy specimens. KEGG pathways showing a Benjamini-Hochberg FDR corrected *p*-value < 0.05 were considered to be enriched.

The B cell receptor signaling pathway was also enriched in the set of down-regulated genes found during severe infection, together with antigen processing and presentation (Figure 4A). On the other hand, the set of up-regulated genes in severely infected turbot yielded enriched functions related to ribosome, oxidative phosphorylation, complement and coagulation cascades and glycolysis/gluconeogenesis (Figure 4B).

**Figure 5:**
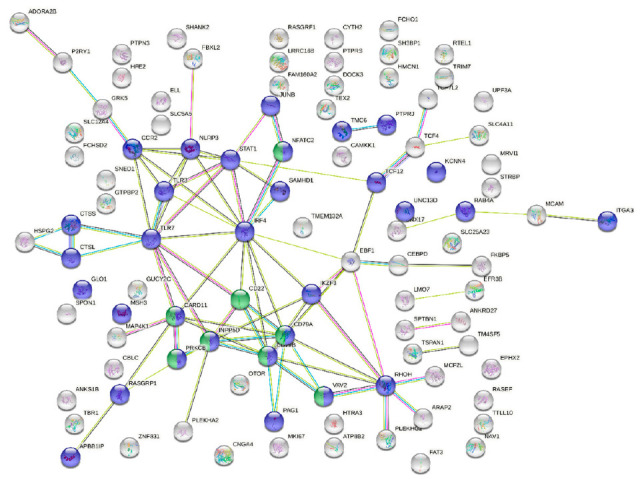
Protein–protein interaction (PPI) network built by STRING database (PPI enrichment *p*-value: 1.97 × 10^−9^), using as input data the differentially expressed genes shared between turbot with moderate and severe enteromyxosis in comparison to controls. Blue nodes denote proteins implicated in immune system process (GO:0002376). Green nodes denote proteins implicated in the KEGG B cell receptor signaling pathway (hsa04662). Nodes represent proteins, while connecting lines denote interactions between two proteins. Different line colors represent the types of evidence used in predicting the associations: gene fusion (red), gene neighborhood (green), co-expression (black), gene co-occurrence (blue), experimentally determined (purple), from curated databases (teal), text-mining (yellow) or protein homology (lilac).

The blood transcriptome of moderately and severely infected turbot shared 143 DEGs (Table S3, Figure S2). These genes were further analyzed by constructing protein–protein interaction networks using STRING. A total of 104 matches were found against human proteins (Table S3), with a final number of 99 nodes, excluding the redundant transcripts (Figure 5). The KEGG pathway and gene ontology (GO) term enrichment analyses performed by STRING on these proteins detected overrepresentation of the B cell receptor signaling pathway and of several GO terms related to the immune response (Table S3). In this regard, a network connecting 40 nodes was found, which showed several proteins involved in immune system processes, including eight acting in the B cell signaling pathway (Figure 5).

## 4. Discussion

In this study, the transcriptomic profile of turbot blood was analyzed in healthy and diseased fish. Increasing the knowledge about this tissue is essential to gain a deeper understanding of the fish immune system and its response to diseases.

Similarly to other fish and terrestrial species, erythrocytes represent the most abundant fraction of blood cells in turbot, around 94% of the total [22]. The primary function of red blood cells is the body transport of oxygen and carbon dioxide, mostly accomplished by the iron-containing metalloprotein hemoglobin. Therefore it is expected that the predominant fraction of transcripts found in turbot blood transcriptome corresponds to this protein, as also observed in other species [23,24,25,26]. The relevance of hemoglobin synthesis in erythrocyte physiology would also explain the large number of genes encoding ribosomal proteins and the enrichment of the KEGG pathways related to ribosome, spliceosome and RNA transport found in the blood transcriptome of healthy turbot. This high rate of protein synthesis demands an above-average bioenergetic need, reflected by the overrepresentation of the oxidative phosphorylation pathway. We recall that oxidative phosphorylation is a metabolic pathway that constitutes the main source of cellular energy in the form of adenosine triphosphate (ATP) [27].

Mammalian, mature erythrocytes lack a cell nucleus and organelles, an evolutionary specialization that permits carrying more hemoglobin and enhances their flexibility and ability to traverse through capillaries [28]. However, non-mammalian erythrocytes are nucleated and contain organelles in their cytoplasm. Hence, they possess the ability to modify their transcriptome and, by reason of their abundance, even genes with low expression in erythrocytes can be important at the organismal level [14]. There is increasing evidence of the contribution of teleost erythrocytes to several immune functions, such as antigen presentation, leukocyte activation or immune cytokine production [11,12,24,29]. In this study, the blood transcriptome of healthy turbot indicated that antigen presentation is also one of the main functions of erythrocytes, with the presence of MHC-I molecules among the top expressed genes, *mhc class Ia chain* and *beta-2-microblobulin*. On that account, the KEGG pathway related to antigen processing and presentation was enriched in comparison with the full transcriptome background, together with the proteasome and phagosome pathways, both involved in processing exogenous antigens for their presentation at the cell surface [30,31,32,33].

The blood transcriptome of *E. scophthalmi*-challenged fish did not exhibit significant changes in the specimens classified as incipient and slightly infected. This observation can be related to the long pre-patent period of enteromixosis [15], and the fact that subtle changes occurring in the early phase of the infection are perhaps not easily detected in a large-scale transcriptomic approach. On the other hand, none of the several diagnostic methods applied to blood samples divulged the presence of *E. scophthalmi* in any sampling points, which is strong evidence against the previously hypothesized parasite dissemination via the bloodstream [18,34,35].

In fish with moderate infection, the vast majority of the DEGs found in comparison to controls were down-regulated. The functional enrichment analysis of these genes unveiled several overrepresented functions related to both innate and adaptive immune response: chemokine, B and T cell signaling pathways, as well as the “focal adhesion” pathways, which can be connected to leukocyte diapedesis [36]. This transcriptomic profile suggests that the triggering of a proper host immune response to the infection is inhibited or depressed during the disease process. The only DEG related to immune response exhibiting an enhanced expression in these fishes was CCAAT/enhancer-binding protein delta (*cebpd*). From studies in humans, it has been shown that the up-regulation of *CEBPD* is mainly associated with inflammatory stimuli [37], and this is one of the DEGs that appeared in moderately infected fish, as well as in those severely infected. Its activation might be interpreted as an early sign of the inflammatory phenomena that will occur with the progression of the disease.

In this sense, up-regulated genes were more numerous than down-regulated ones in fish with severe infection, and many of them were related to the activation of innate immunity. In addition to *cebpd*, another two members of the C/EBP family were up-regulated, *cebpb* and *cebpg*, which are also transcription factors involved in innate immunity [38]. Furthermore, the KEGG pathway related to the complement system was enriched, and other proinflammatory molecules [39] manifested increased expression, such as *interleukin 1b*, *interleukin 8*, *CC chemokine ligand 1*, *vascular endothelial growth factor*, *lysozyme C* and leukotriene- and prostaglandin-related genes (Table S2). These evidences of innate immunity activation were also supported by the enrichment of the metabolic pathways of oxidative phosphorylation and glycolysis/gluconeogenesis, which might be interpreted as an increment of energy needed for the immune response. Particularly, up-regulation of glycolysis is a critical step during immune cell activation, which results in a faster production of ATP compared to oxidative phosphorylation, and is relevant for the synthesis of macromolecules involved in cell activation and proliferation, and for the generation of the antimicrobial respiratory burst [27,40]. The deployment of the innate immune response, as well as the correspondent synthesis of effector molecules [41,42], would also explain the enrichment of the “ribosome” KEGG pathway. Other up-regulated genes to be highlighted are those potentially involved in protection from the deleterious effect of the inflammatory response, related to antioxidant enzymes (*glutathione peroxidase*, *thioredoxin* and *glutathione S-transferase*) [43], anti-proteases (*leukocyte elastase inhibitor*) [44] and negative regulation of cytokine signal transduction (*suppressor of cytokine signaling 2* and *3*) [45].

By contrast, turbot with severe infection also showed several down-regulated genes involved in the immune response; particularly, this set of genes was functionally enriched in the B cell receptor signaling pathway and antigen processing and presentation. Severely and moderately infected fish shared 143 DEGs. The protein–protein interaction network built, and thus indicated, a connection among 40 genes, mostly related to immune response and down-regulated in comparison with control fish, including eight genes involved in the B cell receptor signaling pathway, which was significantly enriched in both groups. Interferon regulatory factor 4 (Irf4) appeared as the hub molecule of this network and has a direct relationship with the genes acting in the B cell signaling pathway. Indeed, its gene expression is mainly restricted to immune cells, where it is a key factor in the regulation of differentiation and is required during the immune response for lymphocyte activation and the generation of immunoglobulin-secreting plasma cells [46]. Interestingly, transcripts related to immunoglobulin heavy and light chains were down-regulated in moderately infected fish, similarly to the heavy chain in severely infected ones.

Two members of the toll-like receptors (TLRs) family, Tlr3 and Tlr7, were also included in this network. This family of genes, encoding receptors that recognize pathogen-associated molecular patterns (PAMPs) and endogenous molecules related to tissue injury, called damage-associated molecular patterns (DAMPs), is evolutionarily conserved and plays a major role linking innate and adaptive immunity [47,48]. Particularly, Tlr3 and Tlr7 are intracellularly localized in the endoplasmic reticulum (ER), endosomes, and lysosome, and are known to be nucleic acid sensors. Their activation induces the synthesis of interferon (IFN) type I and IFN-stimulated molecules [49]. Down-regulation of two IFN-stimulated genes was detected in severely infected fish, *interferon-inducible protein Gig1* and *interferon-induced very large GTPase 1* [50,51], the latter in common with moderately infected fish. Interestingly, another down-regulated gene found in the blood of fish with severe enteromyxosis was *mhc1*. A significant down-regulation of MHC-I and IFN-related genes was previously reported in the kidney and spleen of turbot during advanced stages of enteromyxosis, and it was related to dysregulated interactions between innate and adaptive immunity, possibly contributing to the high susceptibility of turbot to the disease [52].

Previous hematological studies have disclosed that the percentage of circulating lymphocytes decreased progressively in *E. scophthalmi*-infected turbot along the disease course, whereas there was a rise in granulocytes [53]. Likewise, investigations on the kidney and spleen during enteromyxosis showed decreasing numbers of immunoreactive cells to IgM and down-regulation of immunoglobulin-related genes in heavily parasitized turbot [52,54]. The results of blood transcriptomics are then in accordance with those observations, indicating that humoral immunity is ineffective in turbot against enteromyxosis [16,19]. Further, the activation of the innate immunity reflected by the up-regulated genes of severely infected fish is consistent with the increase in the percentage of circulating granulocytes and of the respiratory burst previously reported [53]. All in all, the results of the study support the hypothesis of pathogenetic mechanisms involving the triggering of the inflammatory response not being accompanied by a proper coordination between innate and adaptive responses [15].

## 5. Conclusions

The analysis of the blood transcriptome provided valuable insight into the physiology of this tissue in healthy fish, pointing towards a relevant role of turbot erythrocytes in antigen processing and presentation, in accordance with recent findings in other fish species.

Blood transcriptomics in *E. scophthalmi*-infected fish indicated a delayed response in turbot against enteromyxosis, having no significant gene expression changes in the first stages of the disease. By contrast, we found evidence of immune response inhibition during moderate and severe infection, particularly of B cell signaling and IFN-related pathways, and only an innate response linked to inflammation appeared to be activated in advanced infection. These results are consistent with those of previous studies reporting the presence of an exacerbated inflammatory response not accompanied by a proper development of the adaptive immune response. This response would finally have a negative impact on the fish, resulting in their inability to stop the infection from progressing. Blood transcriptomics is therefore a reliable tool to assess the physiopathological status of turbot.

The development of specific cell markers, the technology for separating and purifying different cell populations in fish and, specifically, single-cell transcriptomics, will help to fully understand the immune functions of the blood and of other tissues. Further studies need to be addressed to unravel the existence of biomarkers of early *E. scophthalmi* infection, possibly increasing the sampling points and sample size at those stages. These studies can pave the way for the use of blood as a non-lethal sample for the early diagnosis of fish health problems through its transcriptomic profile.

## Figures and Tables

**Table 1:** Top 50 transcripts found in the blood of healthy turbot based on their TPM (transcripts per million) values.

Gene Annotation	Mean TPM
*beta-type globin*	224,710.66
*hemoglobin subunit alpha-1*	215,830.97
*alpha-type globin*	107,927.82
*mhc class Ia chain*	22,249.08
*prostate stem cell antigen*	13,256.81
*golgin subfamily a member 5*	6589.07
*mhc class Ia chain*	5201.32
*40s ribosomal protein s30*	4296.66
*ferritin, heavy subunit*	4142.98
*ribosomal protein s11*	3925.73
*ribosomal protein, large, p0*	3478.63
*elongation factor-1 alpha*	3273.46
*40s ribosomal protein s20 isoform 2*	3060.86
*beta-2-microglobulin*	3005.52
*mhc class Ia chain*	2821.07
*40s ribosomal protein s14*	2600.65
*40s ribosomal protein s2*	2514.28
*receptor for activated protein kinase c*	2422.57
*60s ribosomal protein l21*	2416.24
*ferritin, middle subunit*	2399.21
*elongation factor 2-like*	2350.56
*5-aminolevulinate synthase, erythroid-specific, mitochondrial*	2211.96
*40s ribosomal protein s9*	2074.03
*ribosomal protein l6*	2067.13
*muscle-specific beta 1 integrin binding protein 2*	2052.37
*60s ribosomal protein l13*	2027.93
*cytochrome c oxidase subunit 1*	1997.73
*elongation factor 1-alpha*	1944.72
*nudc domain-containing protein 2*	1825.37
*aldehyde dehydrogenase family 16 member a1*	1817.64
*60s acidic ribosomal protein p1-like isoform x1*	1798.65
*heat shock cognate 71 kda protein*	1798.03
*bifunctional methylenetetrahydrofolate dehydrogenase/cyclohydrolase*	1787.53
*60s ribosomal protein l27*	1745.03
*receptor expression-enhancing*	1735.88
*coxsackievirus and adenovirus receptor*	1729.71
*60s ribosomal protein l17*	1717.93
*tubulin beta-2c chain*	1695.68
*40s ribosomal protein s23*	1651.28
*immunoglobulin light chain*	1635.95
*trichohyalin-like*	1581.69
*60s ribosomal protein l4-b*	1537.29
*thioredoxin-interacting protein*	1524.53
*60s ribosomal protein l9*	1521.54
*60s ribosomal protein l8*	1497.61
*myosin ic heavy chain-like*	1496.90
*tubulin beta-1 chain*	1476.30
*40s ribosomal protein s13*	1471.83
*40s ribosomal protein s7*	1410.55
*60s ribosomal protein l5*	1408.27

## Data Availability

Publicly available datasets were analyzed in this study. Raw sequencing data have been deposited in NCBI’s Short Read Archive (SRA) under BioProject ID PRJNA703783.

## Supplementary Materials

The following are available online at https://www.mdpi.com/article/10.3390/ani11051296/s1, Figure S1: Principal components analysis of control, moderately and severely infected turbot by *Enteromyxum scophthalmi*, based on blood transcriptome data. Figure S2: Heatmap of 143 genes differentially expressed in the blood of turbot with moderate and severe enteromyxosis when compared to controls (FDR < 10%). Normalised expression values for each gene have been scaled from −3 to 3 by subtracting the mean and dividing by the standard deviation. Table S1: Blood transcriptome of healthy turbot and KEGG enrichment analysis using the whole turbot transcriptome as background. The genes with a TPM (transcript per million) >50 are included. Table S2: Differentially expressed genes in turbot blood during enteromyxosis and correspondent KEGG enrichment analyses. List of genes showing significant differential expression (FDR *p*-value < 0.05) in turbot blood with moderate and severe enteromyxosis when compared to controls. In both groups, KEGG analyses were performed separately for up- and down-regulated genes. Table S3: Differentially expressed genes found in common in the blood transcriptome of turbot with moderate and severe enteromyxosis. Results obtained using STRING v11 on this gene set, including mapping against human proteins, Gene Ontology term (biological process) and KEGG pathway enrichment analyses.
