# Supplementary figures and images for: Blood Transcriptomics of Turbot Scophthalmus maximus: A Tool for Health Monitoring and Disease Studies
Source: Animals (Basel). 2021 Apr 30;11(5):1296. doi: 10.3390/ani11051296 (PMC8147184; doi:10.3390/ani11051296)

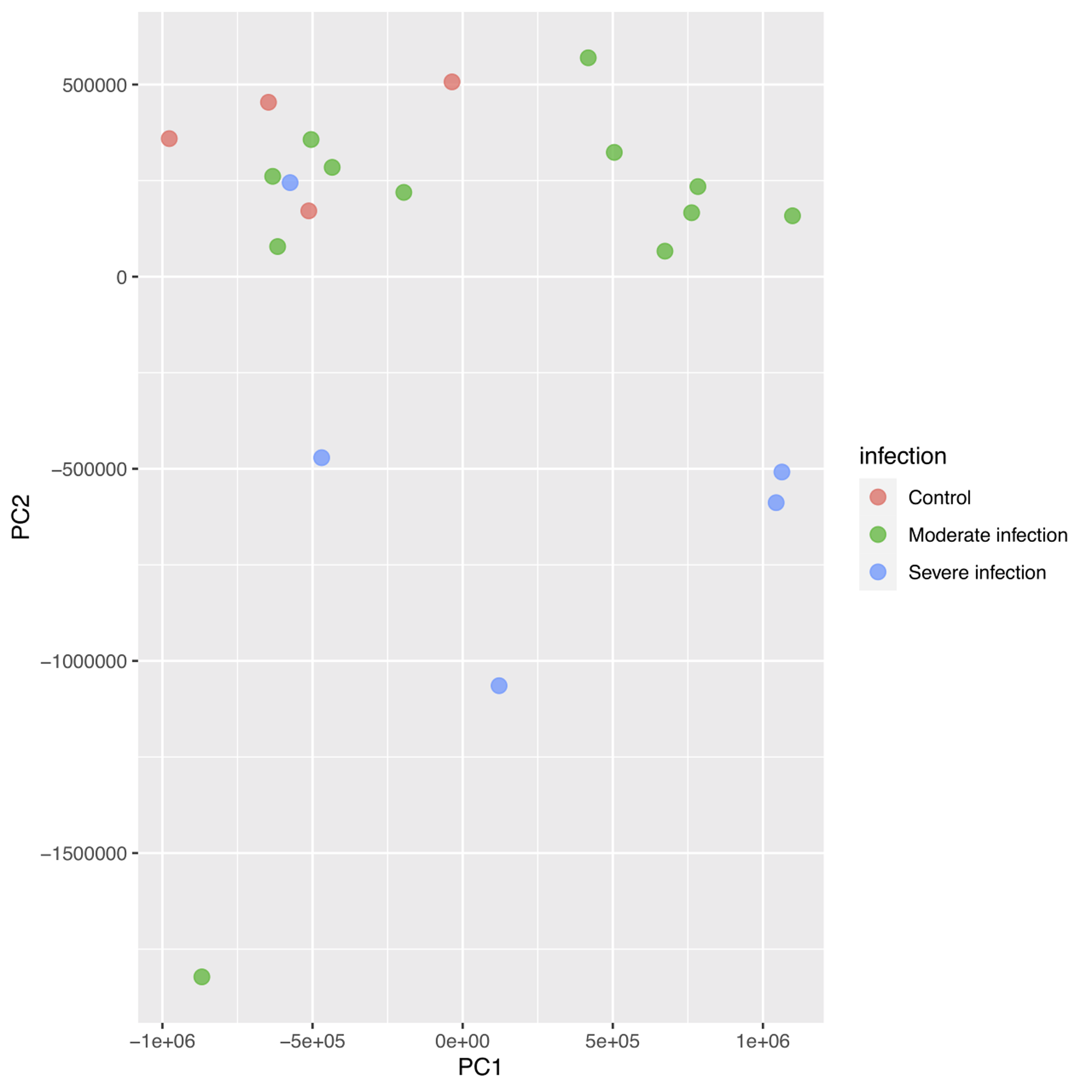

Supplement: Supplementary file 1 [file animals-11-01296-s001.zip › Figure S1_PCA.tif]

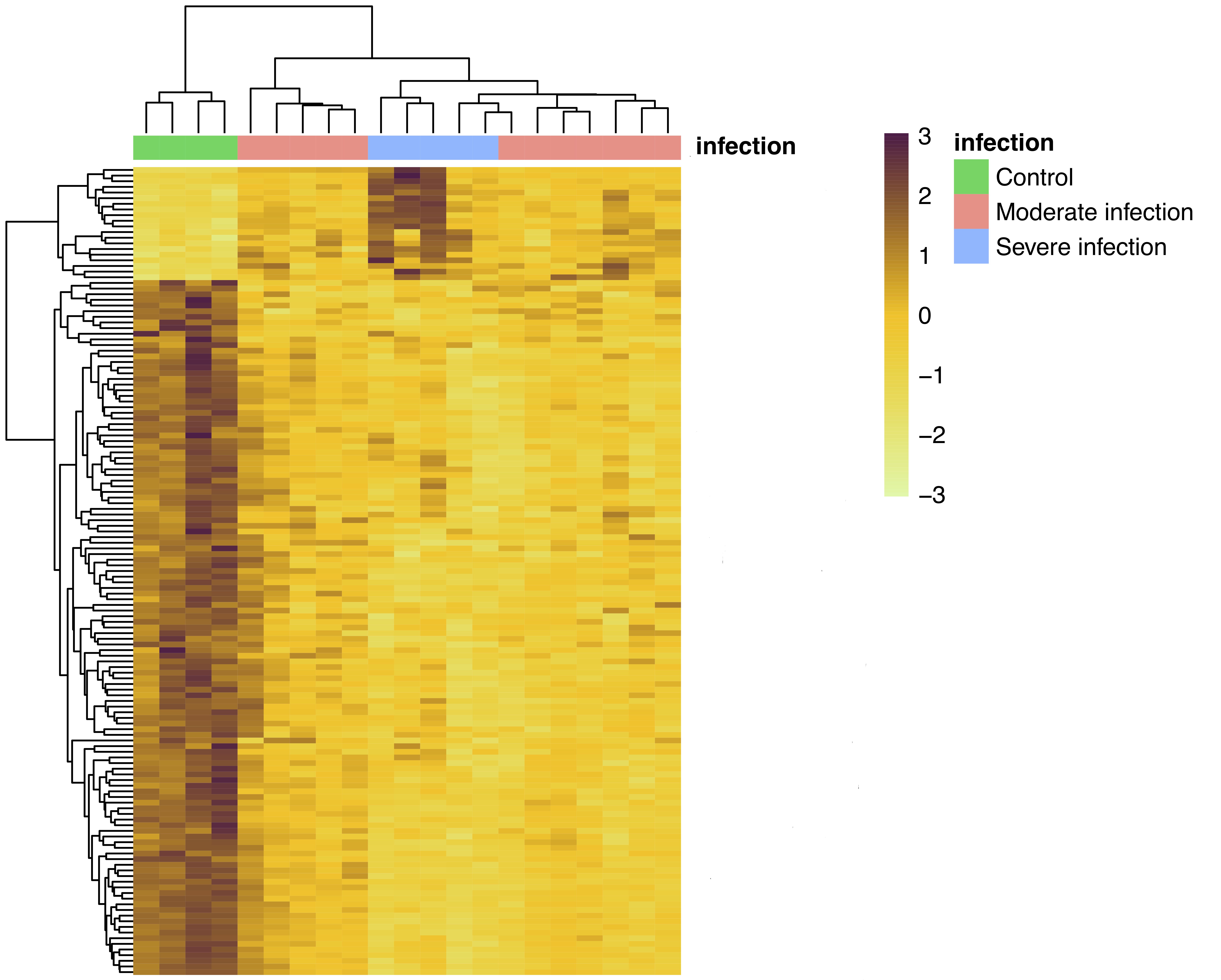

Supplement: Supplementary file 1 [file animals-11-01296-s001.zip › Figure S2_Heatmap.tif]
